# Bacteria dynamics and its correlation with chemical composition changes in tobacco leaves during flue curing

**DOI:** 10.1007/s00253-025-13598-9

**Published:** 2025-09-24

**Authors:** Yunfei Sha, Mengqian Zhou, Demin Liang, Jie Yu, Yanjiu Bi, Qiansi Chen, Huina Zhou, Xianchao Shang, Da Wu

**Affiliations:** 1 Technology Center of Shanghai, Tobacco Group Co. Ltd, Shanghai, 201315 China; 2 China Tobacco Gene Research Center, Zhengzhou Tobacco Research Institute of CNTC, Zhengzhou, 450001 China; 3 College of Plant Protection, Shandong Agricultural University, Taian, 271099 China

**Keywords:** Tobacco leaves, Flue curing, Bacterial community, Chemical composition, Correlation analysis

## Abstract

Microorganisms play a vital role in tobacco growth and processing, yet their correlation with chemical composition during flue curing is understudied. This study examines the dynamic changes in chemical components and bacterial communities during flue curing of lower, middle, and upper leaves. Several key constituents showed distinct changes across leaf positions and curing stages. Xanthophyll decreased during the yellowing stage, while neutral fragrance substances increased after yellowing, especially in middle leaves. Sucrose and proline showed a predominant increasing trend throughout the entire curing process. Nicotine in lower leaves was lowest, and significantly increased by flue curing. Bacterial community analysis revealed higher alpha diversity in lower leaves, dominated by *Proteobacteria*, with *Pseudomonas* and *Acinetobacter* prevalent in lower and middle leaves, and *Aeromonas* in upper leaves. The flue-curing process, especially the yellowing stage, significantly altered bacterial community structure. PICRUSt (Phylogenetic Investigation of Communities by Reconstruction of Unobserved States) analysis revealed stage- and leaf position-dependent variations in predicted microbial metabolic functions, with predominant involvement in amino acid, lipid, and carbohydrate metabolism pathways. Specific bacteria, like *Flavobacterium*, were significantly associated with multiple chemical components, suggesting their potential roles in chemical changes of tobacco leaves during curing. These insights highlight the complex interplay between chemical composition and bacterial dynamics, offering opportunities to optimize the curing process for enhanced tobacco quality.

**Supplementary Information:**

The online version contains supplementary material available at 10.1007/s00253-025-13598-9.

## Introduction

Tobacco is a significant cash crop, widely cultivated globally, and plays a crucial role in the economic development of many countries (Appau et al. 2020; Xu et al. 2019). However, freshly harvested tobacco leaves often exhibit various quality issues, including impurities, irritants, and undesirable flavors (Wang et al. 2015; Yu and Gong 2009). To enhance their quality and produce dry tobacco leaves with optimal physical properties and chemical compositions (Andersen et al. 1988; Morin et al. 2004), the leaves must undergo processing stages, such as flue curing.

All along, research on tobacco flue curing primarily focuses on the changes in chemical composition, particularly those components that are closely related to quality. Based on the variations in these chemical components and the physical state of the tobacco leaves, the process of flue curing can be sequentially divided into three distinct stages: yellowing, color fixation, and stem drying (Zheng et al. 2017; Gong 1994). The degradation of starch predominantly occurs during the yellowing and color fixation stages, with a gradual increase in total sugar and reducing sugar content. Protein content also decreases over the process of flue curing, while the content of free amino acids correspondingly increases (Liu 2008). The degradation of chlorophyll and carotenoids during flue curing is closely related to changes in leaf color, and their degradation products, such as 3-oxo-alpha-ionol and megastigmatrienone, are important aromatic compounds in tobacco (Wang et al. 2012; Yao et al. 2010).

Besides the chemical compositions, it has been reported that the curing process significantly influences the microbiome of tobacco leaves (Chen et al. 2020). Initially, research was often conducted by isolating and culturing microorganisms followed by identification and counting. A study by Gong et al. (2010) indicated that the number of bacteria during the curing process shows an increasing trend at the yellowing stage followed by a decrease at later curing stages. In contrast, Yao (2010) reported that the number of bacterial microorganisms showed an overall decreasing trend during the curing process of tobacco leaves from different leaf positions. The microorganisms identified in these studies ranged from a few to several dozen, and with the advancement of sequencing technology and the reduction of sequencing costs, many more microorganisms, especially those non-culturable or difficult to cultivate, have been discovered and identified. Ding et al. (2023), using 16S rDNA sequencing, identified 1,783 operational taxonomic units (OTUs) from tobacco leaves during the curing process and found that the bacterial community diversity showed an increase at the yellowing stage and then decreased subsequently throughout color fixation and stem drying stages. Hu et al. (2021), using IonS5XL high-throughput sequencing technology, found that the diversity of the bacterial community on the surface of tobacco leaves gradually decreased during the curing process, with the main changes reflected in the relative abundance of *Proteobacteria*, *Actinobacteria*, and *Bacteroidetes*. Yang et al. (2023), through 16S rDNA sequencing, found that no significant changes occurred in the dominant phyla of *Proteobacteria*, *Actinobacteria*, and *Firmicutes* upon flue curing. These studies conducted in-depth analyses of the changes in bacterial communities during the curing process and obtained some microorganisms that may be related to curing conditions and stages. However, reports on the impact of bacterial community structure on the chemical composition of tobacco leaves during flue curing are still rare, and the positional variations in bacterial communities across tobacco leaves have been largely overlooked, making it difficult to recognize and identify key bacteria that may affect the intrinsic chemical changes in the tobacco leaves.

In this study, we investigated the positional and temporal variations of bacterial communities and chemical compositions in tobacco leaves during flue curing, establishing statistically significant associations between specific bacterial taxa and the transformation of key phytochemicals. This research provides a data foundation and theoretical framework for optimizing tobacco flue curing techniques and for analyzing the physiological and biochemical mechanisms underlying the development of tobacco quality.

## Materials and methods

### Tobacco leaves, flue curing and dynamic sampling

*Nicotiana tabacum* L. Zhongyan 100 were growing in the field of Dongzigou village in Jiaxian of Henan Province, China. Mature leaves were sequentially taken from the lower (L, the 4th-5th leaves from bottom), middle (M, the 9th-10th leaves from bottom), and upper (U, the 14th-15th leaves from bottom) positions of tobacco plants at harvest (T1) on July 30, Sep 6, and Sep 14, 2022, respectively. To achieve better tobacco curing quality, the maturity of leaves at different positions was defined according to the rules for the curing technique of flue-cured tobacco of China (GB/T 23219–2008). Briefly, for the lower leaves at maturity, the leaf color is greenish-yellow with a white midrib. For the middle leaves at maturity, the leaf surface is light yellow with white and shiny midribs and lateral veins. For the upper leaves at maturity, the leaf surface is pale yellow with slight yellow spots.

Tobacco flue curing is conducted in an intelligent tobacco curing facility. The target dry bulb temperature is set at 38℃ during the Yellowing stage, gradually increasing to 55℃ during the color-fixing stage, and maintained at 68℃ for 18 h during the dry-rib stage. During flue curing, leaves are collected at 2 days of flue curing (at yellowing stage, T2), 4 days of flue curing (at color-fixing stage, T3) and the end of flue curing (T4), respectively.

The samples, collected from T1 to T4, were divided into two aliquots. One aliquot was flash-frozen in liquid nitrogen and then freeze-dried for chemical composition analysis. The other aliquot was transported to the laboratory at a low temperature (4 °C) for microbial extraction. Three replicates were set for each aliquot.

### HPLC analysis

Xanthophyll, chlorogenic acid, and scopoletin were measured based on HPLC analysis according to previous description (Yang et al. 2024b). Briefly, for xanthophyll analysis, 2 g of freeze-dried tobacco leaf powder was extracted with 25 mL of 90% (v/v) aqueous acetone using ultrasonication for 30 min. The extract was filtered through a 0.45 µm membrane, and 10 μL of the filtrate was injected into an HPLC system (Agilent 1100, USA) for quantification. For chlorogenic acid and scopoletin analysis, 50 mg of tobacco leaf powder was extracted with 10 mL of 50% (v/v) aqueous methanol by ultrasonication for 20 min. Subsequently, 10 μL of the filtered extract was analyzed using the same HPLC system.

### Silanized GC-FID analysis

Proline, nicotine, sucrose, glucose, and fructose in tobacco leaves were measured by a silanized GC-FID method. A mixture of 0.5 mL n-dodecane internal standard solution and 0.5 mL BSTFA-TMCS (99:1) derivatization reagent was added to a vial containing 20 mg freeze-dried tobacco leaf powder. After vortexing for 30 s, the mixture was extracted at 70 °C for 30 min, and then centrifuged at 13,670 g for 10 min. The supernatant was transferred to a 2 mL chromatographic vial for further analysis.

For gas chromatography (GC) analysis, Rtx-1 capillary column (30 m, 0.25 mm ID, 0.25 µm) was employed. The injection volume was set at 1 μL, with a split ratio of 20:1, and the inlet temperature was maintained at 280 °C. Helium was used as the carrier gas at a constant flow rate of 1.5 mL/min. The temperature program was as follows: initial temperature of 125 °C, held for 5 min; ramped at 2.5 °C/min to 210 °C, then increased at 10 °C/min to 300 °C, and held for 15 min.

The flame ionization detector (FID) was set as follows: temperature at 300 °C; hydrogen flow rate at 40 mL/min; air flow rate at 400 mL/min; helium flow rate at 15 mL/min; signal acquisition set at 50 Hz with a sampling interval of 0.004 min.

### LC-GC/MS analysis

Neutral fragrance metabolites, including megastigmatrienone, 3-oxo-alpha-ionol, and 3-hydroxy-β-damascone, were analyzed by a LC-GC/MS method. For each sample, 0.2 g freeze-dried powder was weighed into a 15 mL thick-walled centrifuge tube and extracted by 5 mL extraction solvent (mixture of methyl tert-butyl ether and n-hexane with a volume ratio of 1:1) and 0.2 mL of an alpha-ionone internal standard solution (11.2 µg/mL). The mixture was vortexed for 1 min, allowed to stand overnight, vortexed again, and then centrifuged at 1,510 g for 5 min. The supernatant of 1 mL was transferred into a chromatographic vial for analysis.

A Styragel HR 0.5 (4.6 mm I.D. × 300 mm × 5 µm, Waters) column was used for high-performance liquid chromatography (HPLC) analysis. Mobile phase: dichloromethane; flow rate: 0.25 mL/min; injection volume: 10 µL; column temperature 30 ℃. The diode array detector (DAD) was set at detection wavelengths of 238, 254, and 320 nm. The LC cutting range was from 11.1 to 12.1 min.

Gas chromatography was performed using a DB-5MS column (30 m × 0.25 mm × 0.25 µm). The column temperature was initially set at 39℃ for 14 min, then ramped to 200℃ at a rate of 5 °C/min, and finally increased to 290℃ at a rate of 20 °C/min, held for 5 min. The helium carrier gas flow rate was 1.2 mL/min, and the solvent evaporation flow rate was 20 mL/min. The analysis column was interfaced with the pre-column at 12.9 min and separated from the pre-column at 50 min.

The parameters of the mass spectrometer were as follows: EI ionization mode, ionization energy 70 eV; ion source temperature 230 ℃; mass range 50–350 m/z; transmission line temperature 280 ℃; solvent delay 25 min; monitoring mode: full scan.

### Microbial genome extraction and 16S rRNA sequencing

For microbial genome extraction, approximately 20 g of tobacco leaf samples were cut into small pieces and immersed in 400 mL of sterile PBS solution. The microorganisms attached to tobacco leaves were collected by filtering through a 22 μM membrane after shaking at 200 rpm for 2 h. Subsequently, a soil nucleic acid extraction kit (Shanghai Omega Company) was utilized to extract microbial genomic DNA.

For 16S rRNA sequencing, the V4 region of the 16S rRNA gene was amplified by PCR using the primers 515 F (5′-GTGCC AGCMGCCGCGGTAA-3′) and 806R (5′-GGACTACHVGGGTWTCTAAT-3′) as suggested by Caporaso et al. (2011). The library construction was performed using the NEB Next® Ultra™ II FS DNA PCR-Free Library Prep Kit (New England Biolabs, USA, Catalog #: E7430L), and library sequencing was conducted on the NovaSeq 6000 platform.

### Bacterial community analysis and statistical analysis

Microbial community analysis was performed on high-quality sequencing data (Q30 > 98.67%; see Table S1 for complete processing statistics) processed through Novogene’s MAGIC Platform (https://magic.novogene.com/) using Kraken2 with default parameters. Taxonomic classification was conducted against the Genome Taxonomy Database (GTDB release 207) via the same platform. Alpha diversity and beta diversity were calculated using R vegan (v2.6–10). PICRUSt2 (v2.5.0) was used to predict KEGG pathways from ASV tables, normalized by 16S copy number. For comparative analysis of bacterial communities across leaf positions, samples from all curing stages (T1–T4) were pooled by position (upper/middle/lower; *n* = 12 per position) to examine positional effects independent of temporal variation.

For statistical analyses, chemical-microbe correlations were calculated using CNSknowall (https://www.cnsknowall.com/) for Pearson’s rank correlation. Tukey’s HSD post-hoc test (R package ‘emmeans’, v1.11.2) based on linear mixed-effect model (R package ‘lme4’, v1.1–36) was used for analyzing chemical composition differences. Normality of alpha diversity indices was assessed by Shapiro–Wilk test (R package ‘rstatix’, v0.7.2). Non-normal data were analyzed by Kruskal–Wallis test followed by pairwise Wilcoxon tests, while normal data used ANOVA followed by Tukey’s HSD. Beta diversity was evaluated by PERMANOVA (permutation multivariate analysis of variance) based on Bray–Curtis distances with vegan::adonis2 (999 permutations) and visualized via PCoA. All graphs were generated using ChiPlot (https://www.chiplot.online/) with ggplot2-based templates or R ggplot2 (v3.5.1).

## Results

### Chemical components in tobacco leaves show dynamic changes during flue curing

**Fig. 1:**
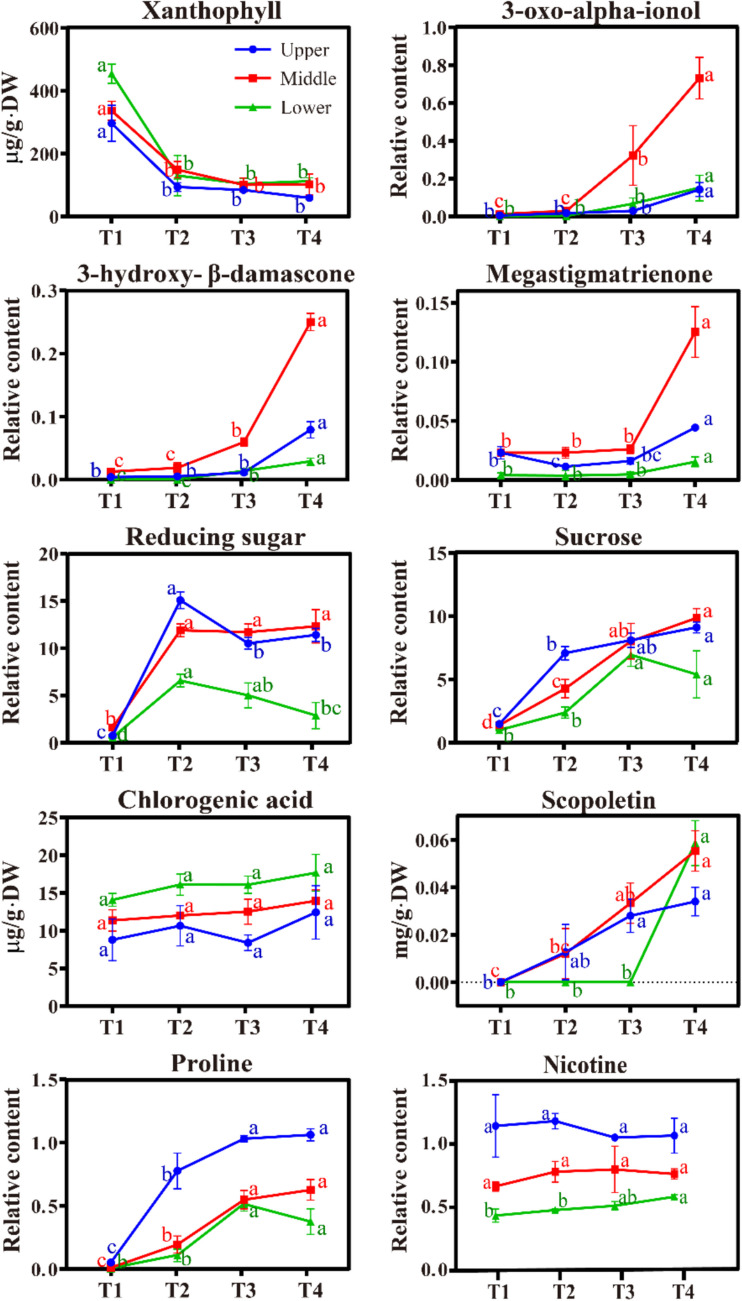
Dynamic changes of several key metabolites in tobacco leaf with curing process. T1–T4 represent the four sampling periods, harvest, yellowing, color-fixing, and the end of flue-curing, respectively. The lowercase letters “a, b, c, d” indicate the results of the significance test (*p* < 0.05) from the analysis of variance (ANOVA)

The dynamic changes in several key chemical components related to tobacco quality were measured and analyzed, as shown in Fig. 1 and Table S2. Xanthophyll, which is closely linked to tobacco leaf color, decreased sharply during the yellowing stage (T2) and then stabilized at low levels. At harvest (T0), xanthophyll levels exhibited a progressive decrease from lower to upper leaves. While no significant difference was observed between middle and upper leaves (Table S2), lower leaves showed significantly higher xanthophyll content compared to both upper and middle leaves (*p* < 0.05), aligning with their maturity differences at harvest. Neutral fragrance substances derived from carotenoid degradation, such as 3-oxo-alpha-ionol, 3-hydroxy-β-damascone, and megastigmatrienone, increased after the yellowing stage (T2), with a rapid rise after the color-fixing stage (T3). Middle leaves exhibited higher levels and increments than upper and lower leaves by the end of flue curing (T4).

Reducing sugar (sum of glucose and fructose) surged early in the yellowing stage (T2), and its content in lower leaves showed a steady decreasing trend compared to the relatively slow or irregular changes in middle and upper leaves. Sucrose gradually increased with the flue-curing process, while no significant variations were detected after the color fixation stage (T3). Throughout flue curing, lower leaves had less reducing sugar and sucrose than middle and upper leaves (Table S2).

Chlorogenic acid, a major polyphenol in tobacco, showed an upward trend with curing, but without significant variance (*p* > 0.05) among stages T1 and T4. Flue curing steadily increased scopoletin content for middle and upper leaves, while a sharp increase was observed for lower leaves after the color-fixing stage (T3).

Proline, the main free amino acid in flue-cured tobacco, gradually increased with flue curing. At the end of curing (T4), upper leaves had the highest proline content, followed by middle and then lower leaves, reflecting a positional pattern. Nicotine content was minimally affected by flue curing, with upper leaves having the highest levels, consistent with proline distribution.

### Bacterial community changed with leaf positions

**Fig. 2:**
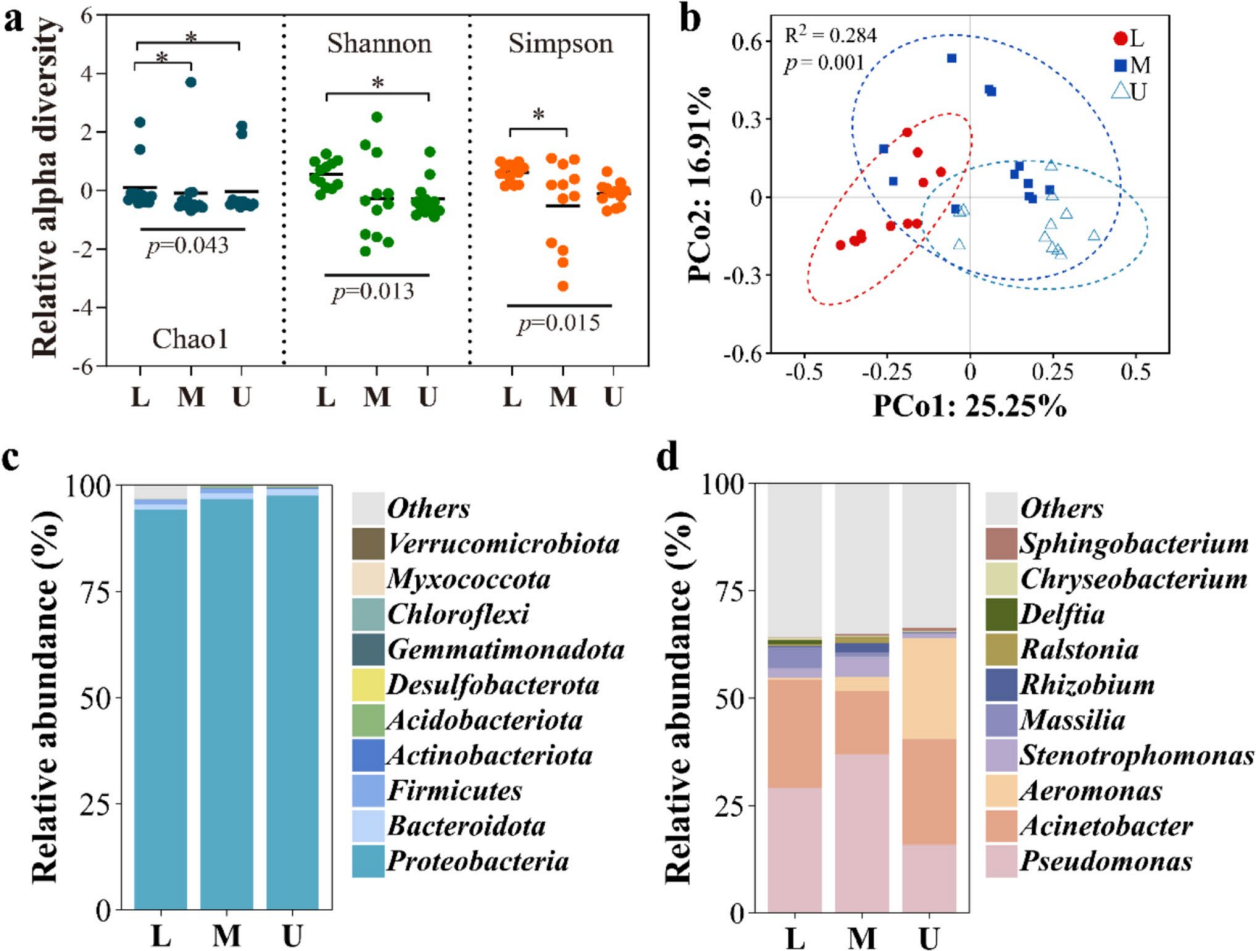
Bacterial community analysis across different leaf positions. L, M, and U denote the lower, middle, and upper leaves, respectively. **a** Alpha-diversity analyses of bacterial communities. *p* values indicate overall significance (Kruskal–Wallis for Chao1/Shannon; ANOVA for Simpson); asterisks denote pairwise significance (Wilcoxon or Tukey’s HSD; *p < 0.05). **b** Principal coordinate analysis (PCoA) based on Bray–Curtis distance, calculated using OUT relative abundance. The relative contribution of different leaf positions to community dissimilarity was assessed using PERMANOVA. **c** Relative abundance of bacterial communities at the phylum level. **d** Relative abundance of bacterial communities at the genus level

During the curing process, from harvest to the end of flue curing, a total of 1195, 1568, and 1535 OTUs were detected in lower leaves (L), middle leaves (M), and upper leaves (U), respectively. As shown in Fig. S1a, 132 OTUs were shared among samples from different leaf positions. To assess overall significant differences among the three leaf positions, based on the normality of each alpha diversity index, the Kruskal–Wallis test was used to analyze Chao1 and Shannon, while ANOVA was used forthe normalSimpsondata. As shown in Fig. 2a, the alpha diversity of bacterial communities among different leaf positions was significantly different at the *p* < 0.05 level for Chao1, Shannon, and Simpson. Pairwise comparison by Wilcoxon test revealed that the Chao1 richness of bacteria was significantly higher in lower leaves, and the Shannon index showed significant differences between lower and upper leaves, with a higher Shannon mean in lower leaves. Pairwise comparison by Tukey HSD revealed a significantly higher Simpson index in lower leaves than in middle leaves. These results suggested a higher alpha diversity of bacterial communities in lower leaves (the panel a of Fig. 2). A principal coordinate analysis (PCoA) based on Bray–Curtis distance indicated slight differences along with the variation of leaf positions (Fig. 2b). Permutation multivariate analysis of variance (PERMANOVA) suggested that leaf position explained 28.4% of the variation in bacterial communities at the *p* < 0.01 level. Further analysis of similarities (ANOSIM) based on Bray–Curtis distance revealed significant differences between different leaf positions at the *p* < 0.01 level (Fig. S1b).

To further understand the differences in bacterial community compositions among different positional leaves, the relative abundances of bacteria at the phylum and genus levels were analyzed. As shown in Fig. 2c and “phylum” sheet in Table S3, the bacterial community on tobacco leaves was predominantly composed of the phylum *Proteobacteria*, with its relative abundance over 94% at all leaf positions. At the genus level (Fig. 2d and “genus” sheet in Table S3), *Pseudomonas* and *Acinetobacter* were the most abundant microorganisms in lower and middle leaves, accounting for over 50% of the bacterial community, while in middle leaves, the relative abundance of *Pseudomonas* was almost 2.5 times that of *Acinetobacter*, contrasting with the comparable abundances of both in lower leaves. The bacterial community composition varied significantly in upper leaves, with *Acinetobacter* and *Aeromonas* being the two most abundant genera, and *Pseudomonas* being the third most abundant genus. These results suggest that leaf positions affect bacterial communities in terms of diversity and structure.

### Bacterial community structure is affected by flue curing

**Fig. 3:**
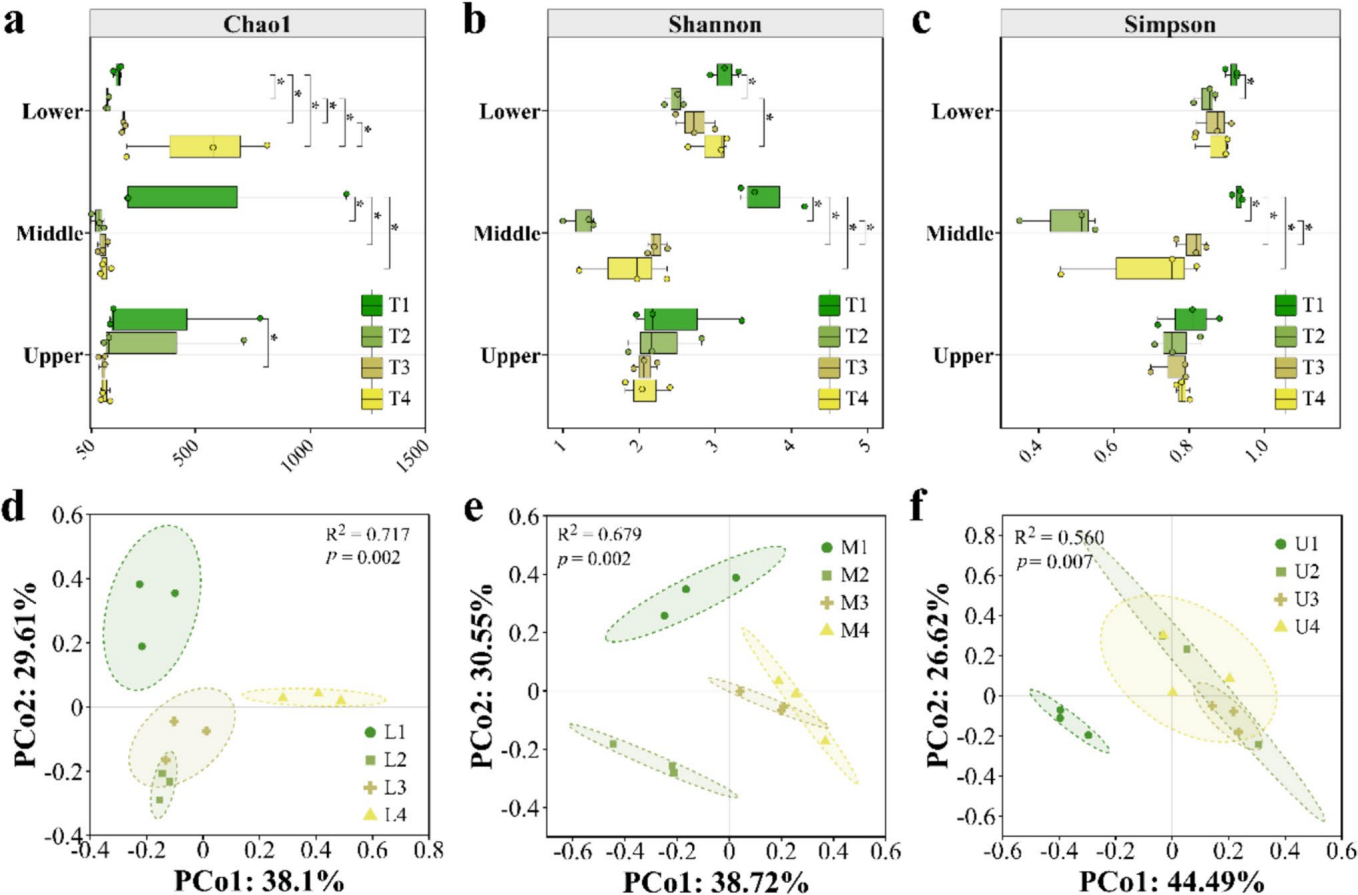
Alpha diversity and principal coordinate analysis (PCoA) of bacterial microbiomes on tobacco leaf surface during flue curing. **a**, **b**, **c** Alpha diversity indices of bacterial communities. **d**, **e**, **f** PCoA based on Bray–Curtis distance. The letters L, M, U, and T represent the lower, middle, and upper leaves, and sampling time, respectively. The numbers 1–4 following the letters correspond to the four sampling stages: harvest, yellowing, color-fixing, and the end of flue curing, respectively

The bacterial community on tobacco leaves varied with the flue-curing process, as shown in Fig. 3 and Fig. S2. For lower leaves, there were 153, 126, 151, and 546 unique OTUs at harvest, Yellowing, color-fixing, and the end of flue curing, respectively, with a total of 65 shared OTUs (Fig. S2a). For middle leaves, there were 1146, 51, 74, and 85 unique OTUs at the four stages, respectively, with a total of 55 shared OTUs (Fig. S2b). For upper leaves, the four stages had 595, 490, 62, and 69 unique OTUs, respectively, with 73 shared OTUs (Fig. S2c). Compared with the harvest stage, the Chao1 richness of bacteria was significantly reduced by flue curing, except for lower leaves, which only showed a significant decrease in Chao1 at the yellowing stage (Fig. 3a). Shannon and Simpson indexes (Fig. 3b and c) of bacteria were also significantly lowered by flue curing for lower and middle leaves, while no significant changes were detected for upper leaves. It was noted that the stage with the greatest change in α-diversity of bacteria on tobacco leaves during flue curing was the yellowing stage. PCoA based on Bray–Curtis distance (Fig. 3d–f) showed that the bacterial community formed distinct clusters according to the flue curing stages, especially the harvest stage, highlighting the distinct nature of the bacterial populations at harvest. PERMANOVA analysis indicated that flue curing had a significant effect on the bacterial community with *R*^2^ = 0.717 (*p* = 0.002) for lower leaves (Fig. 3d), *R*^2^ = 0.679 (*p* = 0.002) for middle leaves (Fig. 3e), and *R*^2^ = 0.560 (*p* = 0.007) for upper leaves (Fig. 3f), respectively. ANOSIM analysis based on Bray–Curtis distance also revealed the significant influence of flue curing on the bacterial community of tobacco leaves (Fig. S2d-2f).

**Fig. 4:**
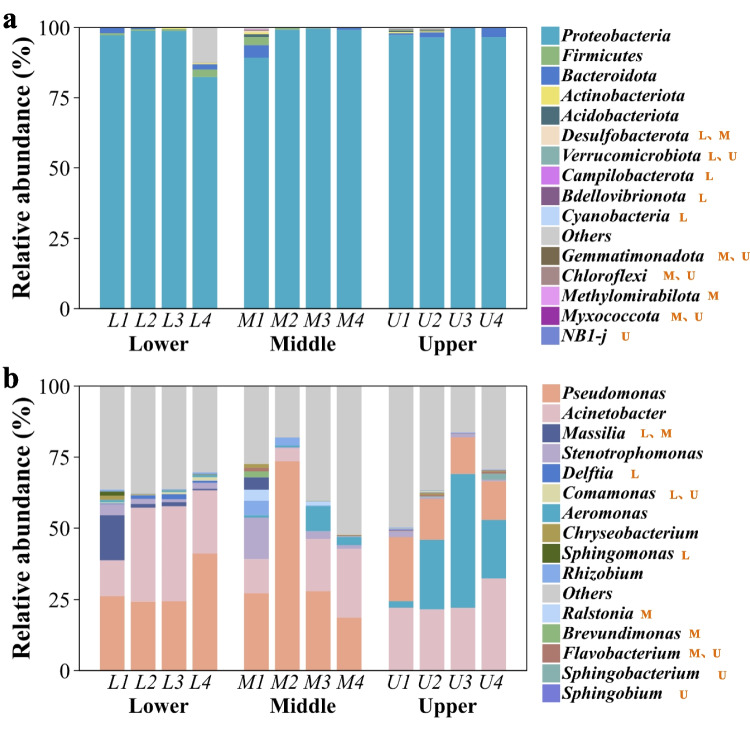
Bacterial communities of lower, middle, and upper leaves during flue curing. **a** Bacterial communities at the phylum level. **b** Bacterial communities at the genus level. The letters L, M, and U represent the lower, middle, and upper leaves, respectively. The numbers 1–4 following the letters correspond to the four sampling stages: harvest, yellowing, color-fixing, and the end of flue curing, respectively. Phyla or genera names followed by orange-colored L, M, and U letters indicate that they are among the top 10 most abundant in the respective leaf position

The compositions of the bacterial community at the phylum and genus level during flue curing were analyzed separately for different samples according to their leaf positions as shown in Fig. 4. At the phylum level, *Proteobacteria* predominated with a relative abundance exceeding 82% for all sampling stages (Fig. 4a and Table S4). Other notable phyla were *Firmicutes* and *Bacteroidota*, which reached over 1% abundance at some stages. In the case of middle and lower leaves, the relative abundance of *Proteobacteria* peaked at the yellowing stage, whereas for upper leaves, it reached its maximum at the color-fixing stage. At the genus level, as depicted in Fig. 4b, the predominant bacteria at the curing process of lower and middle leaves were affiliated with the genera *Pseudomonas*, *Acinetobacter*, *Massilia*, and *Stenotrophomonas*, while the predominant genera of upper leaves were *Pseudomonas*, *Acinetobacter*, and *Aeromonas*. Furthermore, dynamic analysis revealed that the primary bacterial community composition underwent continuous shifts throughout the curing process. As the curing progressed, the prevalence of *Pseudomonas* strains in the upper and lower leaves gradually diminished, whereas in the middle leaves, the relative abundance of *Pseudomonas* experienced a marked increase during the yellowing stage, followed by a gradual decline. Initially, the bacterial communities were dominated by *Pseudomonas*, which later transitioned to a predominance of *Acinetobacter* and *Aeromonas* as the curing process advanced.

### Functional prediction of bacterial communities on tobacco leaf surfaces during flue curing

**Fig. 5:**
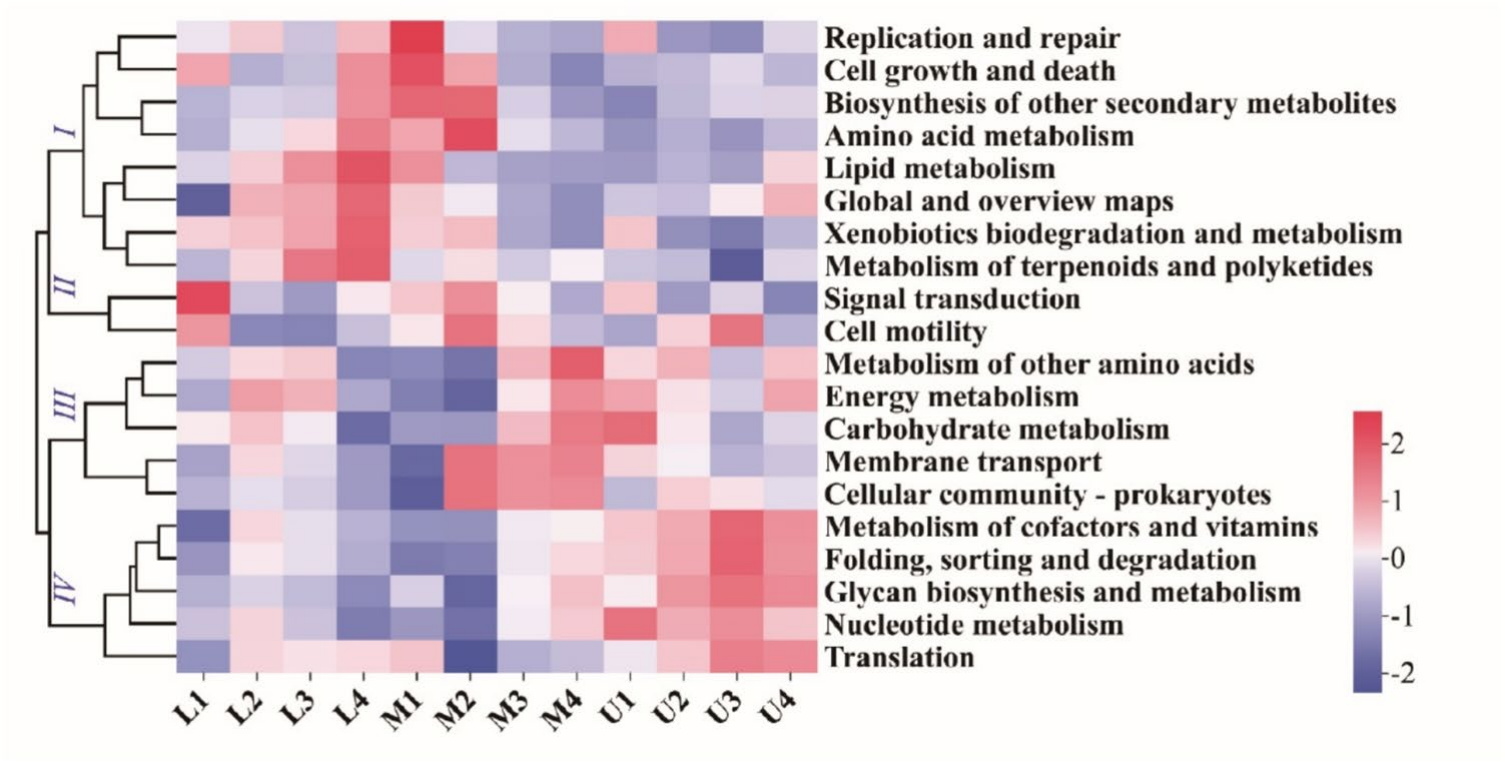
PICRUSt analysis of bacterial community function on tobacco leaf surface during flue curing. The letters L, M, and U represent the lower, middle, and upper leaves, respectively. The numbers 1–4 following the letters correspond to the four sampling stages: harvest, yellowing, color-fixing, and the end of flue curing, respectively. The italicized numerals Ⅰ, Ⅱ, Ⅲ, and Ⅳ denote the four clusters of KEGG functional prediction pathways, scaled across rows

Drawing from bacterial diversity data and PICRUSt analysis, the metagenomic functions of the bacterial communities were predicted based on marker genes, with the detailed outcomes presented in Table S5. The predictions revealed that the most abundant KEGG function at level 1 was metabolism, accounting for about 70% relative abundance (Table S5). At level 2, the dominant bacterial KEGG functions with over 1% relative abundance occupied about 90% of the total abundance during flue curing. The most abundant functions associated with microbial metabolism were identified as amino acid metabolism, metabolism of cofactors and vitamins, and carbohydrate metabolism. No significant differences were observed for the predicted bacterial KEGG functions during flue curing of tobacco leaves from different leaf positions. However, consistent with bacterial diversity, the relative abundance of KEGG functional prediction pathways exhibited dynamic changes across different curing stages of tobacco leaves. As illustrated in panel b of Fig. 5, cluster I, which includes pathways involved in biosynthesis of other secondary metabolites, amino acid metabolism, lipid metabolism, and metabolism of terpenoids and polyketides, showed an increase in abundance in lower leaf samples as curing progressed, while a decrease was observed in middle leaves. Pathways in cluster II were relatively less abundant in the later stages of curing. In contrast, the metabolic pathways in cluster III, such as carbohydrate metabolism, energy metabolism, and metabolism of other amino acids, were predominantly more abundant during the curing process of middle leaves, especially at the end of flue curing. Curing upregulated the relative abundance of pathways in cluster IV, which includes metabolism of cofactors and vitamins, nucleotide metabolism, glycan biosynthesis and metabolism, and translation pathways, in both upper and middle leaves. This indicates that bacterial functional predictions are influenced by both leaf position and the curing process, with the impact of curing on functional abundance varying among different leaf positions.

### Correlation analysis of bacteria and chemical components during flue curing

**Fig. 6:**
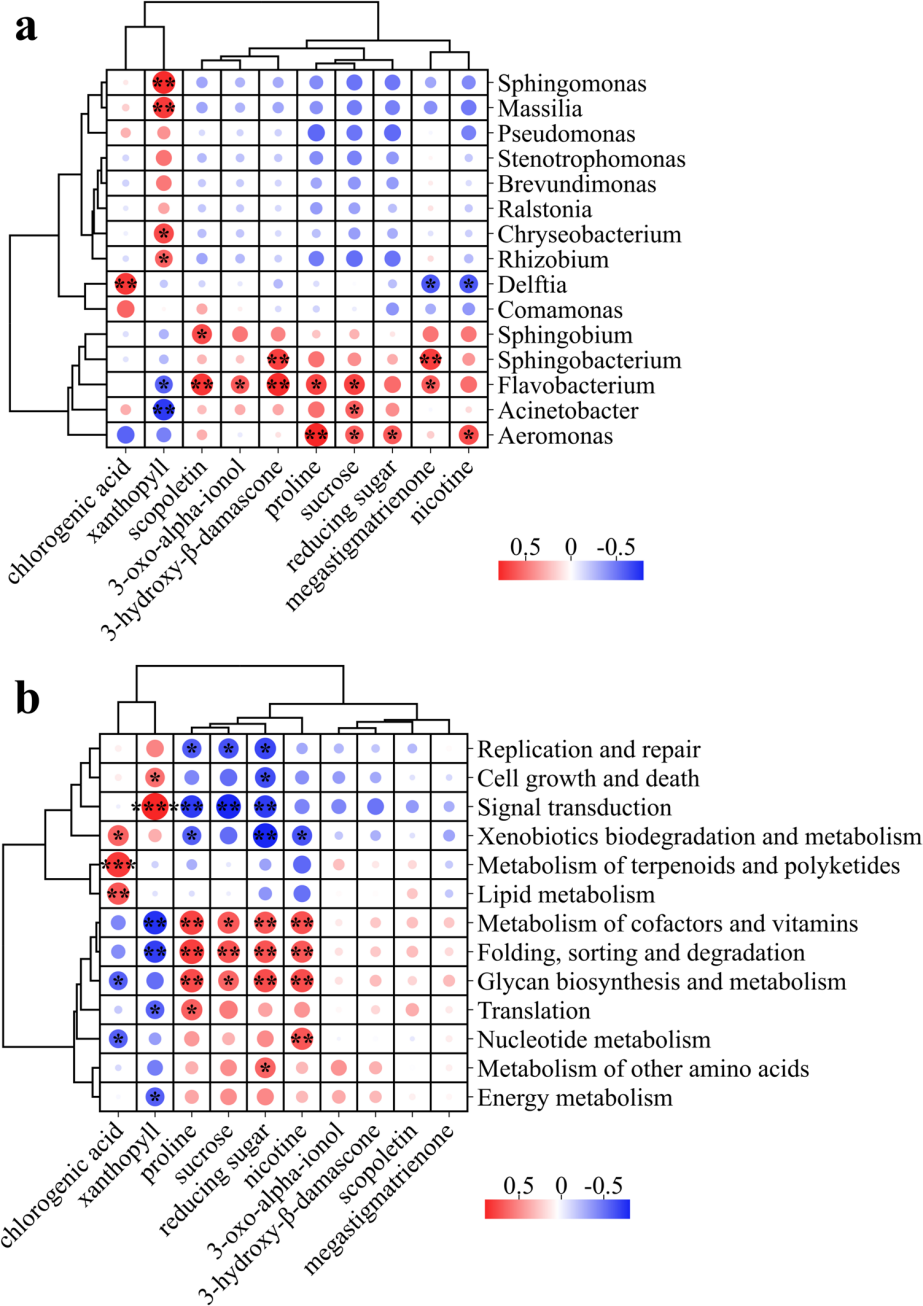
Correlation heatmap of tobacco leaf chemicals with bacterial genera and KEGG functions during flue curing.** a** Correlation heatmap showing the relationship between 10 key chemical constituents in tobacco leaves and dominant bacterial genera. **b** Correlation heatmap of the same chemical constituents with KEGG functions at level 2 predicted using PICRUSt. Asterisks indicate the level of significance based on Pearson correlation analysis: “*” (*p* < 0.05), “**” (*p* < 0.01), “***” (*p* < 0.001), and “****” (*p* < 0.0001)

This study conducted a Pearson correlation analysis to investigate the interplay between chemical components and bacterial communities during the tobacco leaf flue curing process. In Fig. 6, we delineate the relationships between the ten key chemical constituents and dominant bacterial genera as well as dominant KEGG functional pathways. Based on the variations of correlation, the ten key chemicals can be clustered into two groups: one small group containing chlorogenic acid and xanthophyll, and one larger group containing the remaining eight chemicals. The two groups exhibited almost opposite relations with both genera (Fig. 6a) and KEGG pathways (Fig. 6b). Chlorogenic acid is significantly positively correlated with *Delftia*, while xanthophyll is significantly positively correlated with *Rhizobium*, *Chryseobacterium*, *Massilia*, and *Sphingomonas*, and significantly negatively correlated with *Acinetobacter* and *Flavobacterium*. The constituents in the larger group showed varying degrees of positive correlation with *Aeromonas*, *Acinetobacter*, *Flavobacterium*, *Sphingobacterium*, and *Sphingobium*. Additionally, nicotine and megastigmatrienone are significantly negatively correlated with *Delftia*. *Flavobacterium* demonstrated the most significant correlations, exhibiting notable associations with seven key chemical constituents. Figure 6b shows that the three aroma components, 3-oxo-alpha-ionol, 3-hydroxy-β-damascone, and megastigmatrienone, did not yield significant correlations with bacteria metabolic pathways. Proline, sucrose, reducing sugars, and nicotine all showed significant positive correlations with glycan biosynthesis and metabolism, metabolism of cofactors and vitamins, and folding, sorting, and degradation, and significant negative correlations with signal transduction and replication and repair. Chlorogenic acid was significantly positively correlated with lipid metabolism, metabolism of terpenoids and polyketides, and xenobiotics biodegradation and metabolism, while significantly negatively correlated with nucleotide metabolism and glycan biosynthesis and metabolism. Xanthophyll exhibited an extremely significant positive correlation with signal transduction, with a correlation coefficient reaching 0.9, and also showed strong negative correlations with folding, sorting, and degradation, and metabolism of cofactors and vitamins. These results suggest that the dynamics of bacterial communities may significantly influence the chemical composition alterations throughout the curing process.

## Discussion

During the curing process, significant transformations occur in the chemical composition of tobacco leaves, and the role of microorganisms in these transformations was also attracting attention alongside the inherent physiological and biochemical reactions. Utilizing chemical analysis and 16S microbial sequencing techniques, this study identified the dynamic trends of several key chemical constituents and bacterial community structures throughout flue curing. Moreover, a significant correlation was observed between bacterial communities and the key chemical constituents.

### Chemical constituents’ transformation in tobacco leaves during flue curing

The study found a significant increase in sucrose, reducing sugars, and proline content after curing, and a notable decrease in xanthophyll content, which are consistent with previous reports (Gong et al. 2001; Yamaguchi et al. 2013; Yang et al. 2024a, b). The significant rise in chlorogenic acid in middle leaves and the increased content of scopoletin in middle and upper leaves are also in agreement with the findings of Yang et al. (2024a). This study’s findings indicate no significant change in nicotine content during curing of middle and upper leaves, aligning with the results of Chen et al. (2024). While some studies (Amin et al. 1980) suggested a decrease in nicotine content after curing, others (Yang et al. 2024a) argue for a significant increase during the curing process. The discrepancy of nicotine content changes through flue curing may be related to the leaf positions or mature states by harvest, for we detected a significant increase in lower leaves and a decreasing trend in upper leaves after curing (Fig. 1). It is generally accepted that aroma components such as megastigmatrienone, 3-hydroxy-β-dihydrodamalone, and 3-oxo-alpha-ionol are degradation products of carotenoids. This study observed a sharp decline in the main carotenoid component, xanthophyll, during the yellowing phase, while the aforementioned aroma components surged dramatically during the color fixing stage. This may suggest that these compounds are not direct degradation products of carotenoids and require specific time frames or conditions to convert into the final aromatic constituents. The full degradation of carotenoids during the yellowing stage, coupled with the appropriate conversion conditions during the color-fixing stage, are critical for enhancing the aromatic profile of tobacco leaves through the curing process. The concentrations of xanthophyll, chlorogenic acid, proline, and nicotine exhibit distinct changes with leaf positions associated with different maturities, indicating their potential utility in assessing the maturity and quality of tobacco leaves.

### Bacterial community dynamics and functions in tobacco curing process

Previous research on tobacco microorganisms has predominantly concentrated on the interleaf and rhizosphere microbiota during the growth phase of tobacco, or on the microbial shifts during the aging stage and the fermentation process of cigars (Huang et al. 2010; Liu et al. 2021). Besides, Yao (2010) reported a reduction in culturable microbial counts with ascending leaf positions at harvest; Morán Gómez et al. (2013) further demonstrated that the population density of isolated bacterial genera in cured wrapper tobacco progressively decreased from lower to upper leaves. More recently, Ye et al. (2021) employed Illumina sequencing to systematically characterize the bacteria of tobacco leaves at harvest and 1-year storage, revealing significantly higher alpha- and beta-diversity in lower leaves compared to middle and upper leaves. These well-documented vertical stratification of microbial communities across tobacco leaf positions aligns closely with our current findings.

In this study, we found significant differences in both alpha and beta diversity of bacterial communities across different leaf positions. The lower leaves exhibited higher alpha diversity. *Aeromonas*, *Pseudomonas*, and *Acinetobacter* were dominant in the upper leaves, contrasting with the lower and middle leaves where only *Pseudomonas* and *Acinetobacter* were dominant. These differences may be related to the varying environmental conditions and harvesting times of the leaves. Lower leaves, located at the bottom of the plant, are less affected by wind and rain, which may facilitate microbial colonization. Upper leaves are harvested last, and the environmental microbial community may have undergone significant changes by then.

During the curing process, bacterial communities in different leaf positions showed similar dynamic changes. For instance, curing significantly impacted the bacterial community structure on tobacco leaf surfaces (Fig. 3). The significant effect of curing on bacterial community structure aligns with previous studies, but the specific outcomes vary. Hu et al. (2021) suggested that curing reduces bacterial alpha diversity, which is consistent with our findings. Ding et al. (2023) reported an increase in alpha diversity at the yellowing stage, which differs from our results. These discrepancies may be due to differences in tobacco variety, origin, experimental conditions, and sampling times during the curing process. Bacterial communities from different regions may have varying adaptabilities to the high temperature and humidity conditions during the yellowing stage of flue curing.

Consistent with the differences in bacterial community structure and their dynamic changes during curing, the relative abundance of predicted KEGG pathways also exhibited distinct dynamics across leaf positions and curing stages. Amino acid metabolism, lipid metabolism, as well as metabolism of terpenoids and polyketides, primarily increased in relative abundance in the lower leaves as curing progressed. Carbohydrate metabolism, energy metabolism, and metabolism of other amino acids mainly increased in the middle leaves. Metabolism of cofactors and vitamins, nucleotide metabolism, and glycan biosynthesis and metabolism predominantly increased in the upper leaves.

The dynamic changes of bacterial communities, and the substantial internal chemical composition changes occurring in tobacco leaves during flue curing, imply that the bacterial community may correlate with the chemical changes and therefore contribute to the associated curing quality of tobacco leaves. It also provides a theoretical foundation and reference for the application of microbial inoculants in the curing process of tobacco leaves.

Beyond the dynamic shifts in community structure driven by temperature and environmental factors, we observed conserved dominance patterns across all samples. At the phylum level, five dominant taxa (Fig. 4a), *Proteobacteria*, *Actinobacteriota*, *Firmicutes*, *Acidobacteriota*, and *Bacteroidota*, were consistently identified regardless of curing stage or leaf position, while six core genera (Fig. 4b) exhibited stable prevalence, including *Pseudomonas*, *Acinetobacter*, *Rhizobium*, *Stenotrophomonas*, and *Aeromonas* from *Proteobacteria*, along with *Chryseobacterium* from *Bacteroidota*. Comparative analysis with recent 5-year studies on tobacco bacterial communities (spanning growth (Shi et al. 2024), curing (Hu et al. 2021), fermentation (Zhang et al. 2025), and aging processes (Zhou et al. 2020) across cultivars and regions (Hu et al. 2022)) revealed high co-occurrence of *Proteobacteria* and *Actinobacteriota* as dominant phyla, with *Pseudomonas* being the most frequently reported predominant genus. Notably, the common denominators at phylum level in the tobacco phyllosphere microbiota were also observed in soil microbial communities under both field and greenhouse conditions (Cheng et al. 2023; Ahmed et al. 2022; Liang et al. 2024). However, genus-level dominant taxa displayed minimal overlap between phyllosphere and soil. Furthermore, these core bacterial groups were also enriched in both shoot and root endophytic communities across diverse plant species (Huang et al. 2024), suggesting the existence of universal host selection mechanisms in plant–microbe interactions.

### Bacterial metabolism and its relation with tobacco chemical constituents during flue curing

The influence of microorganisms on the transformation of tobacco chemical components has been investigated during the aging and fermentation stages. For instance, in the final fermentation phase of cigars, certain anaerobic bacteria and actinomycetes have been implicated in the synthesis of unique chemical compounds, such as coumarin and vanillin (Liu et al. 2021). The metabolic activities of these microorganisms can impact the levels and distribution of alkaloids and other chemical constituents within tobacco leaves, subsequently influencing the quality of tobacco products (Huang et al. 2022). It has been documented that specific microbial enzymes can break down nicotine, sugars, and other substances into simpler metabolites (Jiang et al. 2022; Ruijssenaars and Hartmans 2001), which not only foster microbial growth but also reduce the levels of harmful substances in tobacco leaves, thereby enhancing the quality of flue-cured tobacco.

Additional research has indicated that certain bacterial strains, such as *Sphingomonas *XP, can degrade polyphenols and starch in tobacco leaves (Feng et al. 2019), and the introduction of this strain during the fermentation process can markedly enhance the sensory attributes of tobacco leaves. This study conducted a comprehensive analysis of the interrelationship between bacterial communities and several key chemical components throughout the tobacco curing process. It is observed that the detected ten chemicals were significantly correlated with particular bacterial communities and could be clustered into two groups according to their correlation patterns with bacteria. Notably, chlorogenic acid and xanthophyll formed one group, and xanthophyll showed a significant positive relation with *Rhizobium*, *Chryseobacterium*, *Massilia*, and *Sphingomonas*, while chlorogenic acid was only significantly correlated with *Delftia*. No reports were found presently about the relationship of these genera with xanthophyll or chlorogenic acid. Carotenoids, degraded flavors, 3-oxo-alpha-ionol, 3-hydroxy-β-damascone, and megastigmatrienone showed a significantly positive relation with *Flavobacterium*. Nicotine exhibited a significant negative correlation with bacteria from the genera *Delftia* and significant positive correlation with bacteria from the genera *Aeromonas*. It was reported that nicotine could be degraded by *Delftia* NLG11, one of the gut symbionts from the brown planthopper (Gong et al. 2023), which is consistent with our negative correlation result. Presently, there is no report about bacteria from the genera *Aeromonas* correlation with nicotine metabolism. Reducing sugars and sucrose were significantly positively correlated with *Aeromonas*, *Acinetobacter*, and *Flavobacterium*, which may be a result of these chemicals serving as a direct carbon source for their proliferation. Leaves with higher sugar content tend to have a greater abundance of these microorganisms. Functional analysis of the dominant bacterial genera, such as *Aeromonas*, *Delftia*, and *Flavobacterium*, which are significantly associated with nicotine, chlorogenic acid, and several flavors, could be valuable in the advancement and application of functional bacteria during the tobacco curing process.

Furthermore, the correlation of the ten key chemicals with bacteria related KEGG pathways was also analyzed. No pathway showed significant correlation with 3-oxo-alpha-ionol, 3-hydroxy-β-damascone, megastigmatrienone, and scopoletin. Nicotine, reducing sugar, sucrose, proline, xanthophyll, and chlorogenic acid were significantly correlated with some KEGG pathways, and reducing sugar and proline exhibited the most correlations, with strong correlations with eight and seven KEGG pathways, respectively. This may indicate that these compounds are closely associated with the colonization and growth of surface microbes on tobacco leaves.

## Conclusion

This study provides a comprehensive analysis of the dynamic changes in chemical components and bacterial communities in tobacco leaves during the flue-curing process. Several key chemical components, such as xanthophyll, neutral fragrance substances, reducing sugars, sucrose, chlorogenic acid, scopoletin, proline, and nicotine, which are closely tied to the quality attributes of tobacco, such as color, aroma, and flavor, undergo significant changes during curing, with variations strongly influenced by leaf position. Flue curing led to a reduction in bacterial richness and diversity, particularly during the yellowing stage, and caused distinct shifts in bacterial community composition and predicted functional potential. Key chemical components showed significant relationships with dominant bacterial genera and metabolic pathways. These findings suggest that specific bacterial taxa and their metabolic activities are closely linked to the biochemical transformations occurring during flue curing, highlighting the intricate interplay between bacterial diversity, chemical composition, and leaf position. Future research should focus on elucidating the functional roles of key bacterial taxa and their interactions with chemical components to further enhance the understanding of tobacco curing and its applications in agriculture.

## Supplementary Information

Below is the link to the electronic supplementary material.

ESM 1 Analysis of leaf position differences in bacterial microbiomes. a) Venn diagram of OTUs detected from different leaves; b) Significance analysis of community structure differences by Anoism method. (PNG 149 KB)

ESM 2 Analysis of bacterial community at different stage of flue-curing . Venn diagrams represent the detected OTUs at different curing stages of lower leaves (a), middle leaves (b) and upper leaves (c). Anoism analysis based on Bray-Curtis distance of lower leaves (d), middle leaves (e) and upper leaves (f). Letter L, M, and U represent lower, middle and upper leaves, respectively, and numbers 1-4 after the letters represent the four sampling stages of harvest, yellowing, fixed color and the end of flue curing, respectively. (PNG 240 KB)

ESM 3 (XLSX 15.7 KB)

ESM 4 (XLSX 27.9 KB)

ESM 5 (XLSX 12.4 KB)

ESM 6 (XLSX 14.6 KB)

ESM 7 (XLSX 21.7 KB)

## Data Availability

The raw sequence data reported in this paper have been deposited in the Genome Sequence Archive (Chen et al. 2021) in National Genomics Data Center (CNCB-NGDC Members and Partners 2022), China National Center for Bioinformation/Beijing Institute of Genomics, Chinese Academy of Sciences (GSA: CRA021981) that are publicly accessible at https://ngdc.cncb.ac.cn/gsa.
