# Supplementary figures and images for: Bacteria dynamics and its correlation with chemical composition changes in tobacco leaves during flue curing
Source: Appl Microbiol Biotechnol. 2025 Sep 24;109(1):205. doi: 10.1007/s00253-025-13598-9 (PMC12460388; doi:10.1007/s00253-025-13598-9)

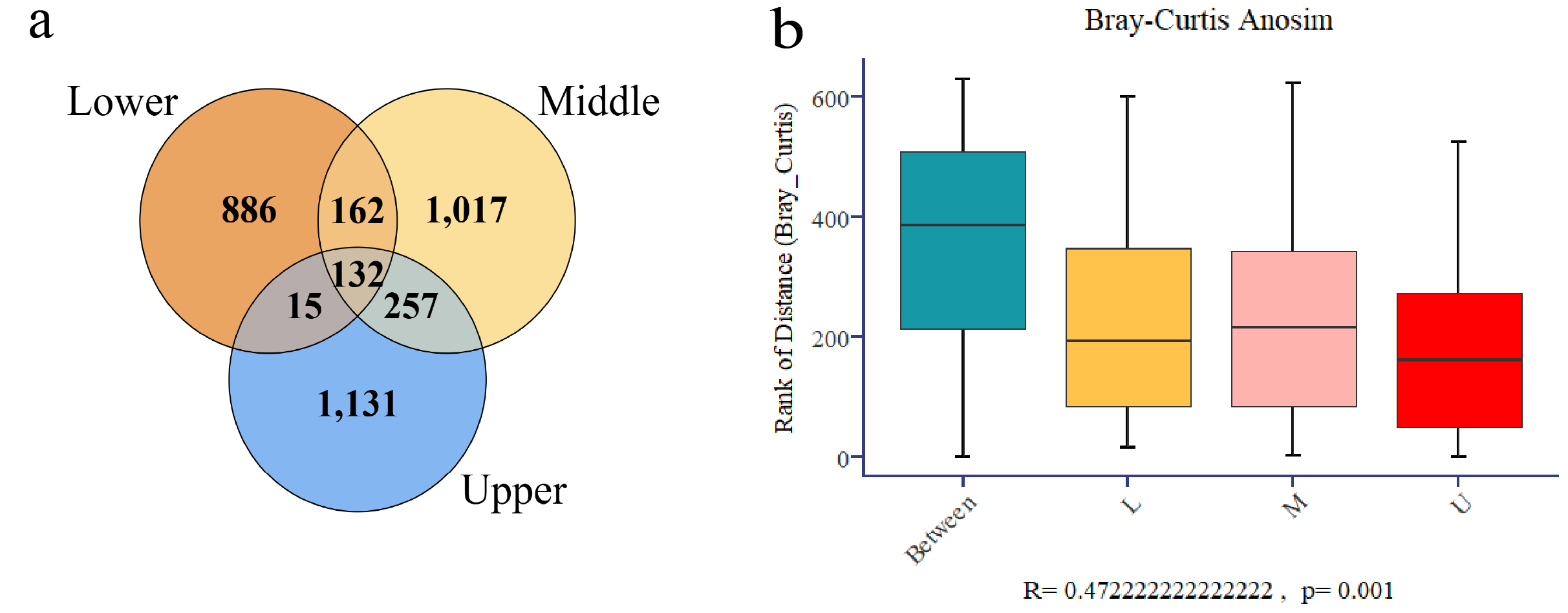

Supplement: Supplementary file 1 — Analysis of leaf position differences in bacterial microbiomes. a) Venn diagram of OTUs detected from different leaves; b) Significance analysis of community structure differences by Anoism method. (PNG 149 KB) [file 253_2025_13598_MOESM1_ESM.png]

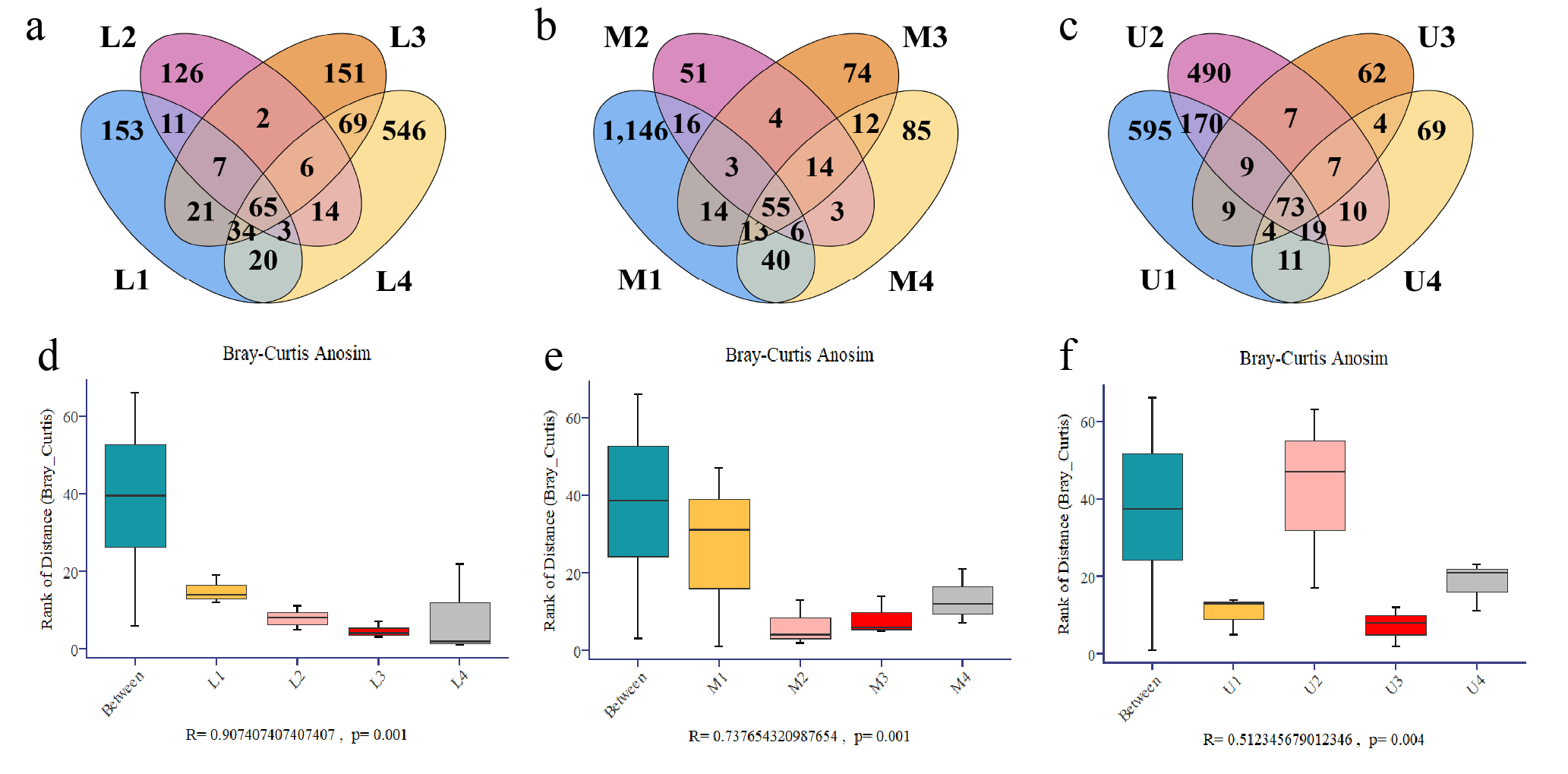

Supplement: Supplementary file 2 — Analysis of bacterial community at different stage of flue-curing . Venn diagrams represent the detected OTUs at different curing stages of lower leaves (a), middle leaves (b) and upper leaves (c). Anoism analysis based on Bray-Curtis distance of lower leaves (d), middle leaves (e) and upper leaves (f). Letter L, M, and U represent lower, middle and upper leaves, respectively, and numbers 1-4 after the letters represent the four sampling stages of harvest, yellowing, fixed color and the end of flue curing, respectively. (PNG 240 KB) [file 253_2025_13598_MOESM2_ESM.png]
